# How Do Healthy Women Perceive the Risk of Breast Cancer? The Role of Illness Perceptions and Compared Risk between Portugal and the U.A.E

**DOI:** 10.3390/ijerph191912923

**Published:** 2022-10-09

**Authors:** Maria J. Figueiras, David Dias Neto, Joao Marôco, Catarina Carmo

**Affiliations:** 1 Department of Psychology, College of Natural Health and Sciences, Zayed University, Abu Dhabi P.O. Box 144534, United Arab Emirates; 2 APPsyCI—Applied Psychology Research Center Capabilities & Inclusion, ISPA—Instituto Universitário, 1149-041 Lisboa, Portugal; 3 William James Center for Research, ISPA—Instituto Universitário, 1149-041 Lisboa, Portugal; 4 School of Psychology, ISPA—Instituto Universitário, 1149-041 Lisboa, Portugal

**Keywords:** breast cancer, illness perceptions, risk perception, compared risk, cultural context

## Abstract

(1) Background: Breast cancer (BC) shows significant epidemiological differences between Eastern and Western countries. These may arise from socio-cultural factors influencing how healthy young women perceive this condition, their risk of getting cancer, and the implications for preventive screening behaviors. In this study, the illness perceptions, individual risk perception, compared risk, and beliefs about preventive behaviors for BC of female university students were compared using an anonymous online survey between a European country (Portugal) and the United Arab Emirates. (2) Method: A structural equation model (SEM) was developed to investigate the hypothetical relationship between illness perceptions and compared risk as predictors of perceived risk for BC. (3) Results: There were significant differences between the study variables. The SEM was invariant, but the differences between regression coefficients in both countries were highly statistically significant. Mediation analyses revealed a significant indirect effect of compared risk on individual risk and a significantly stronger direct effect for the Emirati sample. (4) Conclusions: These findings suggest that cultural research may help to explain factors that may shape social comparison of individual risk characteristics and influence perceived risk. Moreover, providing culturally appropriate strategies to be designed and implemented can promote early detection behaviors for BC.

## 1. Introduction

Breast cancer (BC) is the most common cancer and a leading cause of death in women worldwide [1]. According to the World Health Organization (WHO), in 2020, there were 20.3 million women diagnosed with breast cancer and 685,000 deaths globally. As of the end of 2020, there were 70.8 million women alive who have been diagnosed with breast cancer in the past five years, making it the world’s most prevalent cancer [2]. In Europe, BC accounts for 28% of the total cancers in the WHO European Union [3]. In Gulf Cooperation Council countries, prevalence (cases per 100,000 women) ranged from 26 in Oman to 390.6 in the UAE to 780.7 in Lebanon [4]. Despite these values being below Western countries’ prevalence, there was an overall increasing trend.

The need for preventive strategies at a behavior level implies considering psychological processes in the individual and the cultural background. Concerning the former, cultural background may be one factor in understanding epidemiological data, prevention, and treatment-seeking. In Arab countries, the age of diagnosed women is around ten years earlier than in western countries [5]. This has led some authors to suggest that for some Arab countries, mammography screening should start as early as the age of 30 [6]. This is particularly relevant given the discrepancies between incidence and mortality for BC. For example, Portugal and the UAE have BC incidence rates of 12% and 220.4% and mortality rates of 6% and 120.4%, respectively [7]. Breast cancer has a higher incidence and mortality in the Middle East. This suggests that important differences arise somewhere along the line between prevention and treatment, and some may be due to psychological factors underlying behavior. Little is known about women’s perceptions and other related beliefs about breast cancer and how they can be similar across different cultures [8,9].

In the Arab world, screening programs are relatively new, and adherence to breast screening is low [10]. Among the factors that may influence screening behavior is knowledge about screening amongst women and health care providers, professional recommendations, socio-demographic factors, cultural traditions, beliefs, religious faith, social support, accessibility, and perceived effectiveness of screening [10,11]. The same factors can influence breast self-examination; for example, knowledge about its importance is a predictor of this practice in university students [12].

It is important to consider existing models of health behavior to understand the cultural dimension of epidemiological differences and adherence to treatment. One model that emphasizes patients’ perceptions in explaining patient-reported outcomes is the Common-Sense Model (CSM) of self-regulation (SRM), initially proposed by Leventhal and collaborators [13,14]. According to this model, patients’ personal beliefs about the illness—or illness perceptions—and their emotional response determine how individuals respond to their illness, affecting health outcomes and behavior. Central to this model are illness perceptions organized into components, such as identity, timeline, consequences, control-cure, and cause. Extensive research about the CSM of self-regulation has shown that people actively develop these lay models of illness with a significant impact on their health-related behavior or health outcomes [15].

Most studies on illness perceptions have been conducted with clinical samples. In women with breast cancer, illness perceptions have been found to predict future anxiety and depression [16], a sense of wellbeing [17], psychological distress [18], and fear of recurrence of breast cancer one year after the successful treatment [19]. A meta-analysis of 12 studies with breast cancer patients found that illness perceptions were associated with fear of recurrence, distress, quality of life, satisfaction with medical care, use of traditional healers, and risk perception [20].

Less attention has been given to how healthy women may perceive this condition—namely, the specific beliefs, risk perception, and preventive behaviors they may adopt to prevent BC. With non-clinical samples, illness perceptions have been related to higher levels of distress in women at increased risk for developing breast cancer, contrasting with a comparison sample [21]. These initial findings and the epidemiological data mentioned highlight the need to study healthy young women. Preventive behaviors should start in this age group, particularly in Eastern countries. This supports our aim of exploring sets of beliefs about preventive behaviors and the age-related beliefs associated with BC.

Cultural comparison studies have been made on illness perceptions. For instance, in a cross-cultural comparison between BC patients in The Netherlands and Japan, illness perceptions were significantly different and impacted quality of life [22]. These results show an essential role of illness perceptions in the psychological meaning and emotional reaction associated with illness. A previous study compared 43 Japanese and Dutch patients with breast cancer. Only “concern” showed significant differences. However, they also found lesser variation across cultures than conditions when comparing illness perceptions of breast cancer with other chronic diseases [23]. This previous evidence suggests that further research is needed to understand how illness perceptions and other relevant variables may vary between cultures and influence preventive behaviors and health outcomes.

Another important line of research is the identification of the mechanisms through which illness perceptions lead to behavior. Illness perceptions have been found to relate to risk perception—the perceived risk of personally suffering from a particular illness. A study with healthy women younger than 50 years old found that illness and risk perceptions were predictive of breast cancer worry. Risk perceptions also partially mediated the relationship between illness perceptions and worry [24]. Risk perception has been associated with several preventive procedures, such as vaccination [25] and healthy lifestyles [26]. Risk perceptions for breast cancer tend to be distorted, overestimating risk. When risk perception is according to actual risk, risk perception is related to cancer worry [27]. Since risk perception concerns general risk estimation, it is often thought of as individual perceived risk for an illness and as compared risk (i.e., relative to a similar person in the population). Both measures were included in the present study. This may be an important factor in understanding cultural differences, given expected differences in perceived individual risk and in social comparison [28]. This means that it is essential to consider personal risk estimates and how they relate to other similar individuals’ estimates. Psychological research has recognized that cognitive, affective, and qualitative risk characteristics serve as heuristic tools for classifying and evaluating risk. Also, cultural research has been essential in pointing out cultural factors that influence risk perception [29].

Given the importance of individual risk perception, it is important to assess the relative weight of variables in a multifactorial model that may affect it in different cultural groups. The purpose of this model is to understand how illness perceptions affect risk perceptions in different cultures. As such, the present study has two aims. Firstly, to compare two samples of female university students from a European country (Portugal) and the United Arab Emirates (UAE), regarding (a) illness perceptions, (b) risk perceptions (individual perceived risk and compared risk), (c) beliefs about preventive behaviors regarding breast cancer, and (d) beliefs about age probability of getting breast cancer and the age most likely to undergo an examination to prevent breast cancer. This age group is particularly relevant given the need to anticipate screening behaviors. Secondly, we aim to explore whether illness perceptions and compared risk predict individual risk perception for BC in healthy young women. We hypothesized that illness perceptions about BC are significant predictors of risk perception, and that relationship can be mediated through comparative risk. This model was tested for each cultural group.

## 2. Materials and Methods

### 2.1. Participants and Procedure

A cross-sectional survey on breast cancer was conducted at Zayed University in UAE and ISPA—Instituto Universitário in Portugal. All the students were female within an age range of 18 years old to 25 years old. The survey was implemented on Google forms and sent through campus announcements and social media. 

### 2.2. Measures

The measures included socio-demographic data (age, marital status, and family history), illness perceptions, risk perceptions, beliefs about the age probability of getting BC and undergoing screening, and beliefs about preventive behaviors for BC. The family history of BC was classified into two categories (yes/no).

### 2.3. Brief Illness Perception Questionnaire (Brief-IPQ)

An adapted experimental English version of the Brief-IPQ by Broadbent and colleagues [30] for a non-patient sample was used to measure illness perceptions. The Brief-IPQ has been widely used across several illnesses and different ethnic groups and has been translated into 26 languages showing good psychometric properties across a wide range of studies [31]. An English version was used since the university courses are taught in English. This brief scale has been used in several studies to assess illness perceptions in patients (e.g., [32,33]). The Brief-IPQ consists of nine items rated on a scale from zero (minimum) to ten (maximum). The items assess cognitive perceptions such as effect on life or consequences (item one), duration of illness (item two); control over illness (item three); beliefs about the effectiveness of treatment (item four); and experience of symptoms (item five). Items six and eight assess emotional aspects, including concern about illness and emotional representation of the illness. Item seven assesses the degree of understanding of the illness (coherence). Higher scores indicate more negative beliefs. 

### 2.4. Risk Perceptions (Perceived Individual Risk and Compared Risk)

These measures were adapted from previous studies on risk perception in non-clinical samples [34]. The participants were asked to rate the perceived individual and compared risk of breast cancer on an 11-point scale ranging from zero (no risk) to ten (high risk). The scale was inspired by previous literature on verbal probability expressions used to communicate risk [35,36]. Individual risk perception was measured, asking participants to rate their perceived risk of developing breast cancer in the next ten years. Comparative risk perception was measured, asking participants to rate their perceived chance of developing breast cancer compared to women of the same age. Both types of measures were used to research the correlates of perceived susceptibility [37]. 

### 2.5. Beliefs on Preventive Behaviors for Breast Cancer

A brief questionnaire was designed for this study to measure beliefs about the perceived effectiveness of preventive behaviors for BC. It included seven items concerning health-related behaviors (five items) (such as diet and smoking) and specific behaviors (two items) related to screening for breast cancer which was answered on a scale ranging from zero (strongly disagree) to ten (strongly agree). These items were inspired by several studies about preventive behaviors for breast cancer [38,39].

### 2.6. Age Estimations for Breast Cancer and Screening

Two questions measured beliefs about the age probability of getting breast cancer and undergoing medical examination (screening). These questions were measured on an ordinal scale ranging from age 20–29 to >50, and one response option of “age does not matter.”

### 2.7. Data Analysis

The data analysis was performed in two steps. First, we used a descriptive approach to assess illness perceptions, comparative risk, and perceived individual risk. Differences related to family history were studied using Welch *t*-tests (*p* < 0.05) for large samples under the assumption of the normal distribution of means as per the central limit theorem. These statistical analyses were performed with IBM SPSS version 26 [40]. Power calculations were performed (post-hoc) using Gpower3.1 [41]. The achieved power for comparison between groups ranged from 97% to 99%. Due to the low percentage of married participants, marital status was not considered for analysis. The structural equation model was fitted with the lavaan package (v00.6; [42]) for the R statistical system to probe hypothesized causal relations between illness perceptions and perceived risk.

## 3. Results

### 3.1. Sample Characteristics

The sample included 298 UAE and 344 Portuguese female students from different university courses in both countries. The mean age was 20.9 years old (SD = 10.7) for the Portuguese students and 20.1 years old (SD = 10.7) for the UAE students, ranging from 18–25 years old. Concerning marital status, 98.5% of the Portuguese students and 91.3% of the UAE students are single; 1.5% of the Portuguese students and 8.5% of the UAE are married. Due to the low percentage of married students, this comparison was not included. Concerning the family history of BC, 21.5% of the UAE students and 37.8% of the Portuguese reported it.

### 3.2. Comparison between Groups on the Study Variables

A comparative analysis was performed using a *t*-test for independent samples on illness perceptions, risk perception, and beliefs about preventive behaviors. The results are presented in Table 1.

#### 3.2.1. Illness Perceptions and Risk Perception (Individual Perceived Risk and Comparative Risk)

There were significant differences between the two countries on illness perception dimensions. The UAE students show more positive perceptions about the consequences, timeline, identity, concern, personal control, and emotional representation than the Portuguese students. Concerning perceptions about treatment control and coherence, the UAE students have more negative views (Table 1). Regarding risk perception, there were significant differences between the two groups. The UAE group showed a lower perceived individual and compared risk to the Portuguese group.

#### 3.2.2. Family History of Breast Cancer

There were significant differences in individual risk perception and compared risk concerning family history of breast cancer. The participants with a family history of BC reported an overall higher perceived risk than the other participants. Comparing the two groups of participants with family history of BC, the Portuguese students showed a significantly higher individual perceived risk (*t* (192) = −4.68, *p* < 0.001, d = −0.715) and compared risk (*t* (192) = −3.64, *p* < 0.001, d = −0.556) than the Emirati students.

#### 3.2.3. Beliefs about Preventive Behaviors

There were no significant differences in the general preventive behaviors between the two groups. However, there were significant differences in beliefs about specific breast cancer preventive behaviors, such as mammograms and BSE. The Portuguese students hold stronger beliefs about regular self-examination and routine screening than the Emirati students (Table 1).

#### 3.2.4. Beliefs about Age Probability of Getting BC and Undergoing Screening to Prevent BC

**Figure 1:**
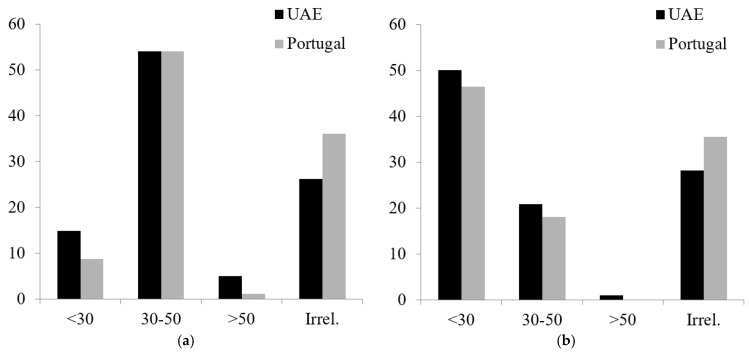
Ages more likely to get breast cancer and to undergo breast examination (percentages for both groups). (**a**) Age of BC onset. (**b**) Age for BC screening. Note: “Irrel.” Refers to “age does not matter” option.

Considering each country, the percentage of the items measuring beliefs about the probability of getting BC and undergoing screening to prevent BC is reported in Figure 1. A chi-square test of independence was performed to examine the association between the beliefs and the two nationalities. The association between these variables was significant for the following items: beliefs about age probability of getting BC in their 20s and 30s and after their 50s, in which Emirati students hold a stronger belief than the Portuguese. Concerning the belief that “age does not matter to get BC,” the association was significant. The Portuguese students hold a stronger belief than the Emirati students (χ^2^(3642) = 18.090, *p* < 0.001). There were no significant associations concerning beliefs about the age to undergo screening for BC and the participants’ nationality.

### 3.3. To Explore Predictors of Individual Risk Perception for BC in Healthy Young Women in Each Country

**Figure 2:**
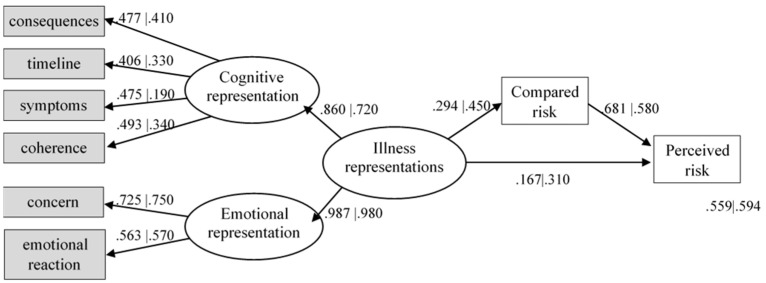
Illness perceptions and compared risk as predictors of individual risk perception for BC. Note. Coefficients on the left are from UAE | Coefficients on the right are from Portugal.

A SEM was developed to investigate the hypothetical relationship between illness perceptions and compared risk as predictors of individual risk perception for BC. The model tested the influence of illness perceptions and comparative risk on perceived individual risk perception in both countries. More specifically, we sought to explore whether comparative risk could mediate the relationship between illness perception dimensions and perceived individual risk. Only the dimensions with significant betas were retained in the model (Figure 2). The model fit was good for both countries (χ^2^(28) = 398.328, *p* < 0.001, CFI = 0.976, TLI = 0.963, RMSEA = 0.040 with 90% CI (< 0.001, 0.067), SRMR = 0.044) and both metric and scalar invariance was observed between countries (∆χ^2^(5) = 6.627, *p* = 0.249 and ∆χ^2^(5) = 8.115, *p* = 0.150, respectively). However, differences between regression coefficients in both countries were highly statistically significant (∆χ^2^(33) = 103.477, *p* < 0.001). For the Portuguese students, the illness representation is a stronger predictor of compared risk (β = 0.446, *p* = 0.003) than for the Emirati students (β = 0.294, *p* = 0.005). The same occurs concerning individual perceived risk (β = 0.314, *p* = 0.006) for the Portuguese students and (β = 0.167, *p* = 0.024) for the Emirati students. However, the compared risk is a stronger predictor of perceived individual risk for Emirati students (β = 0.681, *p* < 0.001) than for the Portuguese (β = 0.578, *p* < 0.001). Concerning the mediation effect, the compared risk is a stronger mediator for perceived individual risk in the Portuguese students (β = 0.258, *p* = 0.003) than for UAE students (β = 0.200, *p* = 0.004).

## 4. Discussion

The present study compared illness perceptions, risk perceptions, and beliefs about prevention for breast cancer and explored predictors of individual perceived risk for BC in two groups of female university students, one from Portugal and one from the UAE. There were significant differences between the two groups of participants concerning some of the components of the CSM. Given the scarcity of studies about BC with healthy women, we can only speculate about these differences. In the present study, the differences found between the groups in the dimensions of the CSM suggest different implications for risk perception. The Emirati participants seem to have a less negative perception of BC than the Portuguese, except for treatment control and comprehension. This suggests that Emirati women have a weaker belief in controlling the illness through treatment and a poorer understanding of the illness. Previous evidence indicated that illness perceptions of healthy women about BC might be influenced by contextual factors regarding, for instance, shared knowledge amongst women and health care providers, cultural beliefs, religious faith, and perceived effectiveness of screening behavior [10]. In addition, the evidence provided by the CSM emphasizes several mechanisms, such as the activation of an illness-related memory or schema (“prototype”), which is based on personal history, knowledge, beliefs about the illness, perceptions of severity, and potential action plans (14). These “prototypes” can be found in patients, and non-clinical samples [21,24,32,43] and may have implications for health-related behavior. Furthermore, the CSM asserts that self-regulation takes the form of a hierarchical structure nested in the self-system (e.g., attitudes and goals); both are nested in the social and healthcare system, which in turn is nested in the broader cultural and ecological context [44]. Therefore, illness perceptions can be influenced by individual, social and cultural factors, influencing decisions, behaviors, and health outcomes. The extent to which illness perceptions of BC are associated with screening rates in each culture should be addressed in future research.

The theoretical framework of illness perceptions and self-regulation has also been applied to understand and predict behaviors related to risk-related beliefs [45]. The present data indicated that a family history of breast cancer is associated with individual and comparative risk perception. This is consistent with previous research [46], in which family history shows an increased risk perception. However, the Emirati students with a family history of BC showed a lower perceived individual and compared risk than the Portuguese. One possible explanation may be related to differences between the countries in reproductive factors. Factors such as younger age at first pregnancy, early breastfeeding, childbearing, and contraceptives [47], can be protective factors for breast cancer [48,49]. Other factors associated with genetics and lifestyle may also contribute to this difference. Possibly, perceptions about social and family support may also reduce the estimates of perceived risk. These are possible explanations that should be further investigated in future research.

The present findings explain how illness perceptions may influence perceived risk for BC in two groups of university students from different socio-cultural contexts. We found that the explanatory model used was invariant. This suggests a general cross-cultural relationship between illness perceptions and risk. However, there were significant differences in the relationship between the variables for each group. These differences may be related to how illness perceptions may serve to generate risk estimates based upon their emotional and cognitive components and contextual factors. The results suggest a higher direct and mediated relationship between illness perceptions and individual risk perception for the Portuguese group. This means that illness perceptions play a more important role in Portuguese women estimating risk. However, the direct relationship between compared risk and individual perceived risk was stronger for the UAE participants. It is important to highlight that the model attained a similar amount of explained variance in both groups. Still, other cultural variables (e.g., religion) may be important in estimating risk regarding the understanding of individual risk perception. This should be addressed in future research.

Considering both groups and the characteristics of each cultural context, the mediation effect of compared risk can possibly be explained by how information about health issues is shared within each context and influences individual risk perception. The mediation effect raises questions concerning how the characteristics of the context may shape social comparison. Cultural research may help explain factors that may influence perceived risk through social comparison of individual risk characteristics. It is possible that compared risk may have a different impact on personal risk perception according to socio-cultural characteristics, which might have implications for preventive behaviors. One important dimension to consider is the individualistic vs. collectivistic focus within a culture. Although much research is needed, there are suggestions that these dimensions may have implications for health outcomes [50]. The collectivistic or individualistic focus differs between Western (e.g., European) and Eastern cultures (e.g., Arab). Comparing different cultures on the psychological processes involved may shed light on the role of these cultural dimensions in risk perception and health outcomes. For example, individually, cultural differences may be reflected in the comparative nature of risk perception. These, in turn, may help to explain health and illness dimensions and subsequent behavior, thus explaining epidemiological differences. Moreover, considering the characteristics of a collectivistic vs. individualistic culture, social, and family factors may play a different role in shaping aspects of early cancer detection, promoting biases by comparison to their close relatives or friends, and influencing individual risk estimation in different ways. The comparison of the behavioral impact of risk estimation in different cultures is an important next step in terms of research. Here, contextual barriers may prove important determinants of preventive behaviors.

The role of the socio-cultural context concerning the accuracy of compared risk deserves further investigation since it may be an opportunity to understand how comparative estimates influence information processing for BC and the possible implications for promoting or hindering preventive behaviors. Furthermore, illness perceptions of BC, namely perceptions of control (personal and treatment), may influence comparative risk in different ways when the characteristics of the context are considered. Since the model in the present study only included individual psychological variables, future research could consider more socially minded factors.

Concerning health implications, no differences were found in beliefs about general preventive behaviors between the participants. This is not surprising given the amount of shared information available around the world about healthy lifestyles and preventive behaviors through different channels, namely social media. However, there were significant differences in beliefs about specific preventive behaviors for BC, namely regular self-breast examination and routine screening (mammogram, clinical assessment). A recent study from Saudi Arabia indicated that a large number of students had inadequate information concerning symptoms of breast cancer, risk factors, and preventive and early detection behaviors [51]. The same authors argued that an educational intervention had a significant effect on raising awareness about breast cancer prevention and suggested that these education programs be introduced in university syllabuses. According to another recent study in the Arab region, women’s knowledge and awareness about BC can be influenced by several factors, such as wrong health practices, social barriers, fear, and social stigma associated with the disease, and may contribute significantly to medical help-seeking behaviors [51].

In the present study, the Emirati participants reported weaker beliefs in screening and self-breast examination (BSE) than the Portuguese. These differences may also have implications for seeking medical help. One possible explanation may relate to factors associated with health-seeking behavior, such as discussing with close relatives or the characteristics of the healthcare provider (e.g., being assisted by a male professional). Another possible explanation relates to different levels of knowledge about specific preventive behaviors for BC. In both cases, these preventive behaviors may involve sensitive issues that may be more relevant in some cultures. Considering that this sensitivity influences perceptions, the results suggest the importance of considering culture in promoting these practices. One may consider that the present findings suggest that the Portuguese participants may be more individually psychologically minded, whereas the UAE participants may be more socially contextually minded. The contrast between Western and Eastern socio-cultural contexts seems to deserve further attention regarding the implications of adopting BC preventive behaviors.

Concerning the beliefs about the age probability of getting BC, the early ages were significantly associated with being Emirati, suggesting the possibility of a specific awareness for this early age, which matches with the epidemiological data from the Middle East countries [52]. The belief about getting BC between the 40s and 50s is independent of nationality, suggesting this belief is according to the available information about BC for this age group. The Emirati participants also showed a stronger belief about getting BC than the Portuguese for the latter age group. However, concerning the belief that “age does not matter,” there was a significant association with the Portuguese participants. This finding suggests that the Portuguese participants may be more aware of symptoms that may occur regardless of age, which may imply a different pattern of beliefs about preventive and help-seeking behaviors for BC than the Emirati participants. These findings also raise questions regarding age as a psychological moderator of perceived vulnerability considering individual factors (self), environmental factors, or behavioral risk-taking [53]. These results are especially worrying given that the age onset in the UAE (as in other Arab countries) is lower than in Portugal (as in other European countries), which is usually associated with a poor prognosis. Special care is needed to raise awareness of BC in Arab countries in a culturally respectful manner to increase preventive practices and early diagnosis.

We would like to acknowledge some limitations. Firstly, the study was conducted on two convenience samples. Selection biases associated with this characteristic may have been different across countries. Nevertheless, the fact that the tested model showed similar results for both samples suggests otherwise, but still, validity issues imply care in extrapolating the results. A reverse sensitivity analysis of the model could have explored the role of individual risk perception as a mediator, being a possible avenue to expand future research. It is also essential to consider that this study focused on specific age groups and women from a background with high education levels. If, on the one hand, this allows culture comparisons by having similar samples in these aspects, on the other hand, it suggests the need for future studies with more diverse and representative samples. Even so, focusing on a specific age group (young women) can shed some light on the psychological factors that might increase screening since a diagnosis of BC at a young age is a known indicator of poor prognosis. In addition, a high level of education may contribute to a better understanding and knowledge about BC, significantly contributing to sharing information for prevention in their communities and families. We were unable to inquire about socio-economic status (SES) given the sensitivity of this topic in the socio-cultural context. Still, we consider that future research should explore the importance of this variable for breast cancer screening.

## 5. Conclusions

Leventhal et al. [14,54] acknowledged the importance of social contextual factors in the CSM and called for their inclusion in future research [55]. The scarcity of work examining CSM in healthy individuals suggests the need to consider the inclusion of socio-cultural and contextual factors as predictors of risk perception for BC and their impact on preventive behavior.

The present findings highlight:

(a) The importance of comparative risk as a mediator between illness perception of BC and perceived individual risk, raising important questions to inform future approaches to assess perceived risk for BC and their influence in adopting protective behaviors.
(b) How social comparison and reduced risk perceptions may undermine the need for screening and breast self-examination.
(c) How the content of illness perceptions may serve as the basis for appraisals of likelihood and severity, influencing perceived individual risk mediated by compared risk.
(d) How demographic characteristics and psychosocial factors can influence perceived risk, suggesting the need to integrate these factors into the design and implementation of future educational interventions.
(e) Interventions must consider the needs and characteristics of the target groups by providing culturally appropriate strategies to promote early detection behaviors for BC.

## Figures and Tables

**Table 1:** Comparison between countries on the study variables.

Variables	UAE	Portugal	*t*-Test	Signif.
**Illness Perceptions** (Range: 0–10)	Mean (sd)	Mean (sd)	(df; value)	*p*-value
Consequences	8.13 (2.20)	9.03 (1.79)	(571.8; −5.64)	<0.001
Identity (symptoms)	6.13 (2.56)	6.66 (2.41)	(640; −2.69)	0.007
Timeline	6.02 (1.79)	6.97 (1.68)	(640; −6.90)	<0.001
Personal control	5.57 (2.36)	6.20 (2.42)	(640; −3.30)	0.001
Treatment control	3.14 (2.30)	2.19 (1.78)	(556.3; 5.79)	<0.001
Concern	6.52 (2.69)	7.88 (2.16)	(568.1; −6.97)	<0.001
Coherence	4.96 (2.53)	4.17 (2.21)	(594.1; 4.18)	<0.001
Emotional representation	6.36 (2.93)	7.20 (2.47)	(584.1; −3.92)	<0.001
**Risk Perception** (Range: 0–10)	Mean (sd)	Mean (sd)	(df; value)	*p*-value
Individual perceived risk	3.43 (2.47)	5.40 (2.50)	(640; −9.99)	<0.001
Compared risk	3.30 (2.50)	4.84 (2.49)	(640; −7.76)	<0.001
**Beliefs about preventive behaviors** (Range: 0–10)	Mean (sd)	Mean (sd)	(df; value)	*p*-value
Healthy diet	7.22 (2.80)	7.65 (2.28)	(572.3; −2.09)	0.037
Non-smoker	7.88 (2.80)	7.86 (2.54)	(640; 0.093)	0.926
Not being overweight	6.88 (2.96)	7.13 (2.66)	(602.3; −1.12)	0.263
Physically active	7.67 (2.65)	7.97 (2.57)	(640; 0.087)	0.931
Breastfeeding	5.28 (3.12)	5.09 (3.15)	(640; 0.783)	0.434
Regular self-breast examination	7.97 (2.57)	9.18 (1.75)	(512.0; −6.83)	<0.001
Routine screening (mammogram, clinical assessment)	7.65 (2.65)	9.04 (1.88)	(525.5; −7.54)	<0.001

## Data Availability

The dataset generated and analyzed during the current study is not publicly available due to the sensitive nature of the topic in this cultural context but is available from the corresponding author upon reasonable request.
